# Nephrology Fellowship Clinician-Performed Ultrasound Curriculum

**DOI:** 10.24908/pocus.v7iKidney.14980

**Published:** 2022-02-01

**Authors:** Nathaniel Reisinger, Nova Panebianco

**Affiliations:** 1 Division of Renal, Electrolyte and Hypertension, Perelman School of Medicine, Penn Medicine; 2 Division of Bedside Ultrasound, Department of Emergency Medicine, Perelman School of Medicine, Penn Medicine

**Keywords:** Clinician-Performed Ultrasound, Medical Education, Lung Ultrasonography, End-Stage Kidney Disease

## Introduction

 Fluid overload (FO) contributes significantly to the development of cardiovascular disease among patients with end-stage kidney disease (ESKD) on hemodialysis (HD), yet remains underappreciated due to limitations of the physical exam 1. Lung ultrasound (US) is an established tool for quantification of FO 2. Previous validation of quantitative lung US among nephrology attendings using a remote web-based lung ultrasound training has been demonstrated 3. Interest in ultrasound education among nephrology fellows is high 4. Clinician-performed ultrasound curricula for nephrology fellows have been described previously, however fellows’ competency in quantitative lung US has not been described 5. In the present study we aimed to assess the current level of knowledge among nephrology fellows as well as the efficacy of a brief training course in quantitative lung US. 

## Methods

For lung ultrasound didactics we employed a web-based, mobile-optimized, app-supported curriculum covering the basics of lung US as well as quantitative lung US. Pre- and post-course short-answer didactic content tests were used to assess learning. Pre- and post-course surveys using a 5-point Likert scale assessed confidence in five domains of clinician-performed ultrasound including psychomotor skills, knobology, image acquisition, lung US image interpretation, and clinical integration. 

Additionally, trainees had a single 15-minute hands-on, one-on-one teaching session with a peer experienced in clinician-performed ultrasound in order to develop psychomotor skills acquisition. Images were obtained using a commercially available, ultra-portable, curvilinear US transducer and tablet computer. Each fellow obtained four 5-second ultrasound clips. A panel of expert ultrasound trainers reviewed images remotely for each fellow and provided individualized feedback on image adequacy and acquisition.

An image bank of 20 previously obtained representative lung US clips was assessed by the same panel and given a B-line score of 0-10. 10 scored clips were used as a training set for the fellows. The other 10 clips were scored by the fellows after the training program. Scores for each fellow were tested against the scores of the panel using interclass correlation coefficients which were confirmed using regression analysis. A Bland-Altman plot was constructed to analyze for bias. Paired two sample means t-test with a one-tailed p value were used to compare pre- and post-test scores.

## Results

Surveys showed statistically significant increases in confidence in five domains critical to clinician-performed ultrasound. On the 5-point Likert scale confidence in psychomotor skills domain increased from a pre-test mean of 2.1 to a post-test mean of 4.3. Confidence in the knobology skills domain increased from a pre-test mean of 1.8 to post-test a mean of 4.1. Confidence in the image acquisition skills domain increased from a pre-test mean of 1.8 to a post-test mean of 3.9. Confidence in the image interpretation skills domain increased from a pre-test mean of 2.1 to a post-test mean of 4.2. Confidence in clinical integration increased from a pre-test mean of 2.7 to a post-test mean of 4.3. In the aggregate, confidence across the 5 skills domains increased from a pre-test mean of 2.1 to a post-test mean of 4.1. P < 0.001 for comparisons. Table 1. On the knowledge assessment, average score increased from 4% before the course to 92% after the course. 

**Table 1:** Clinician-Performed Ultrasound Domain Confidence Assessment. Assessment of confidence of nephrology fellows before and after a brief training course employing a 5-point Likert Scale.

**Skills Domain Assessed **	**Pre-Course Mean**	**Post-Course Mean**	**p-value**
Psychomotor	2.1	4.3	<0.001
Knobology	1.8	4.0	<0.001
Image Acquisition	1.8	3.9	<0.001
Image Interpretation	2.1	4.2	<0.001
Clinical Integration	2.7	4.3	<0.001
Aggregate	2.1	4.1	<0.001

Comparing each fellow’s scored clips to the expert panel’s assessments, interclass correlation coefficients were 0.93, 0.93, 0.97, 0.97, 0.98, 0.95, 0.91, 0.75, 0.84, and 0.82 respectively for each fellow with an average of 0.90, and no scores below 0.75, demonstrating agreement in interpreting B-line scores which was confirmed on regression analysis. Bland-Altman demonstrated agreement with low bias. All user-generated images were reviewed as sufficient for clinical assessment by the expert trainers. 

## Discussion

Our results demonstrate increased nephrology fellow confidence in clinician-performed ultrasound across 5 critical domains following a brief training combining a web-based curriculum and short hands-on peer-mentored training. We showed ready uptake of critical knowledge for obtaining and interpreting quantitative studies. Further, the agreement of fellows’ scores with an expert panel was excellent with high inter-rater reliability and minimal bias. These results expand on the work of Gargani, et al. demonstrating that nephrology fellows can be quickly trained in obtaining and interpreting quantitative lung US. 

## Conclusion

Clinician-performed quantitative lung US can be taught to nephrology fellows quickly and reliably. Further study is needed to determine whether training translates to clinical application and competence.

## Disclosures

None
